# Genetic evidence of aberrant striatal synaptic maturation and secretory pathway alteration in a dystonia mouse model

**DOI:** 10.3389/dyst.2022.10892

**Published:** 2022-12-14

**Authors:** Dhananjay Yellajoshyula, Sunday Opeyemi, William T. Dauer, Samuel S. Pappas

**Affiliations:** 1 Department of Neurosciences, Case Western Reserve University, Cleveland, OH, United States; 2 Peter O’Donnell Jr. Brain Institute, University of Texas Southwestern Medical Center, Dallas, TX, United States; 3 Department of Neurology, University of Texas Southwestern Medical Center, Dallas, TX, United States; 4 Department of Neuroscience, University of Texas Southwestern Medical Center, Dallas, TX, United States

**Keywords:** dystonia, torsinA, TOR1A, DYT1, striatum, cholinergic, synaptic, maturation

## Abstract

Animal models of DYT-TOR1A dystonia consistently demonstrate abnormalities of striatal cholinergic function, but the molecular pathways underlying this pathophysiology are unclear. To probe these molecular pathways in a genetic model of DYT-TOR1A, we performed laser microdissection in juvenile mice to isolate striatal cholinergic interneurons and non-cholinergic striatal tissue largely comprising spiny projection neurons during maturation. Both cholinergic and GABAergic enriched samples demonstrated a defined set of gene expression changes consistent with a role of torsinA in the secretory pathway. GABAergic enriched striatum samples also showed alteration to genes regulating synaptic transmission and an upregulation of activity dependent immediate early genes. Reconstruction of Golgi-Cox stained striatal spiny projection neurons from adult mice demonstrated significantly increased spiny density, suggesting that torsinA null striatal neurons have increased excitability during striatal maturation and long lasting increases in afferent input. These findings are consistent with a developmental role for torsinA in the secretory pathway and link torsinA loss of function with functional and structural changes of striatal cholinergic and GABAergic neurons. These transcriptomic datasets are freely available as a resource for future studies of torsinA loss of function-mediated striatal dysfunction.

## Introduction

DYT-TOR1A (DYT1) is a dominantly inherited dystonia characterized by early onset involuntary abnormal movements and postures (1, 2). TorsinA resides in the endoplasmic reticulum and nuclear envelope lumen, where interaction with cofactors LAP1 and LULL1 promote its ATPase activity (3-9). The DYT-TOR1A disease mutation deletes a single glutamic acid (ΔE) (2), impairing torsinA function (5,8,10-12). The natural history of DYT-TOR1A suggests that processes occurring during development are particularly important for disease pathogenesis (reviewed in (13)) and findings in mouse models suggest that the functions of the *Tor1a* encoded protein torsinA are essential during a developmental critical period but dispensable in adult animals (14). Several CNS developmental processes are altered by torsinA loss of function. In animal models, *Tor1a* deletion or *Tor1a*^*ΔE*^ knock-in disrupts nuclear envelope structure (15, 16) and alters nuclear pore distribution and function (17, 18) during a postnatal CNS developmental period in which neuronal nuclear pore complex biogenesis and insertion is upregulated (19). TorsinA dysfunction causes deficits in secretory processing, protein quality control, and translational control (20-26) and alters synapse formation (27-30), all of which potentially contribute to the altered synaptic plasticity identified in dystonia (31-34). The mechanisms underlying synaptic changes in torsinA deficient neurons are not defined.

Multiple animal models of torsinA dysfunction display aberrant corticostriatal plasticity, including enhanced long term potentiation and decreased synaptic inhibition (24,35-37). Abnormal cholinergic signaling contributes to disrupted plasticity in some DYT-TOR1A models (36) and antimuscarinic compounds improve disease features in some people with DYT-TOR1A (38). Altered striatal plasticity is an early pathophysiological feature. Knock-in mice expressing the *Tor1a*^ΔE/+^ disease mutation exhibit premature long term potentiation, impaired long term depression, and increased AMPA receptor abundance in corticostriatal synapses during early striatal development (29). This converging evidence suggests that diminished inhibitory synaptic function (39) and dysfunction of striatal cholinergic interneurons (ChI) (40) are drivers of dystonia and suggest that processes occurring during development or maturation are critical for dystonia pathogenesis.

To mechanistically explore the relationship between torsinA loss-of-function and synaptic and behavioral change, we modeled DYT-TOR1A dystonia by conditionally deleting torsinA in forebrain inhibitory and cholinergic neurons using Dlx5/6-Cre (41) (“Dlx-CKO mice”). TorsinA is thus deleted from all neurons in the striatum, globus pallidus, reticular thalamic nucleus, and basal forebrain, and from inhibitory interneurons in the cortex and hippocampus in Dlx-CKO mice. Like the natural history of DYT-TOR1A, Dlx-CKO mice exhibit motor dysfunction beginning as juveniles, which worsens with increased handling and is responsive to antimuscarinic treatment (41). During the same juvenile period, a subpopulation of ChI in the dorsolateral striatum selectively degenerate. These findings suggest that Dlx-CKO mice model a link between cholinergic and motor dysfunction (42) believed important in human DYT-TOR1A dystonia (43).

To probe the mechanisms by which torsinA loss alters ChI and surrounding cell types during striatal maturation, we conducted RNAseq analyses on maturing Dlx-CKO striatal ChI somas or surrounding striatum tissue (mainly comprised of spiny projection neuron somas, with a small proportion of GABAergic interneuron somas, glia, and neural processes) isolated using laser microdissection. Striatal cholinergic and non-cholinergic enriched samples from control vs. Dlx-CKO identified a core set of genes enriched in secretory pathway and synaptic function. We further demonstrate abnormal synaptic structure in Dlx-CKO striatum with Golgi-Cox staining of spiny projection neurons. This study identifies a role for torsinA within the secretory pathway and implicates abnormal synaptic structure in the torsinA deficient striatum.

## Materials and methods

### Animals

Animal work described in this manuscript has been approved and conducted under the oversight of the UT Southwestern Institutional Animal Care and Use Committee. Male and female control (Tor1a^Flx/+^) and Dlx-CKO (Dlx5/6-Cre^+^; Tor1a^Flx/−^) mice expressing ChAT(BAC)-eGFP (JAX strain 007902) were generated as previously described (41).

### Laser microdissection and RNA isolation

Brains were harvested at postnatal day 14 (P14) and snap frozen in dry ice-chilled isopentane. 16 μm fresh frozen brain sections were generated with a cryostat, mounted on PET membrane slides, and dehydrated in ethanol and xylenes. Laser microdissection was performed using the ×20 objective of a Leica LMD7 microscope. ChAT-eGFP+ cell bodies (341–524 GFP+ somas per brain) or GFP-negative striatal tissue (1–1.5 million μm^2^ tissue area collected per brain) was laser microdissected and lysed in buffer RLT with 1% β-mercaptoethanol (Qiagen). Total RNA was isolated using a RNeasy-micro kit (Qiagen) and eluted in RNase-free water. RNA quantity and integrity was assessed using an Agilent Bioanalyzer and samples with RIN between 7.6–9.3 were used for RNA sequencing. Samples were derived from the following number of animals: Chi soma *n* = 6 control and *n* = 6 Dlx-CKO; Striatum: *n* = 4 control and *n* = 5 Dlx-CKO.

### RNA sequencing and analysis

RNA-seq was performed using the HiSeq2500 (Illumina) platform in the University of Michigan Sequencing Core. RNA-seq libraries were generated using SmartSeq4 (Clontech). Libraries were quantified and normalized using an Agilent Bioanalyzer and sequenced using the HiSeq2500 High-Output SBS V4 single-end 50 cycle kit. The quality of the raw reads data was checked using FastQC (version 0.11.3). Low quality bases from individual reads were trimmed using CutAdapt. Tuxedo Suite software package was used for alignment, differential expression analysis, and post-analysis diagnostics (44-46). We aligned reads (genome build UCSC mm10) using TopHat (version 2.0.14) and Bowtie (version 2.2.1). We used FastQC for a second round of post-alignment quality control to ensure that only high quality data would be input to expression quantitation and differential expression analysis. We used Cufflinks/CuffDiff (Version 2.2.1) for expression quantitation, normalization, and differential expression analysis. Diagnostic plots were generated with CummeRbund package. We used locally developed scripts to format and annotate the differential expression data output from CuffDiff. Genes were designated as DE if they passed quality control (Cuffdiff/Cufflinks QC test status = “ok”), had Benjamini-Hochberg FDR q-values <0.05, and fold change >1.5 (Tables 1, 2). The raw count data for all replicates are provided in Supplementary Tables S7, S8 and the alignment rates are provided in Supplementary Table S9.

### Gene ontology analyses

Differentially expressed genes identified from RNAseq were further analyzed for the identification of biologically enriched pathways by gene ontology (GO) enrichment analyses using the following web based applications: GENEONTLOGY http://geneontology.org/docs/go-enrichment-analysis/ and DAVID https://david.ncifcrf.gov/home.jsp.

### Golgi-cox staining

Brains from 10 to 14 week old female control (Tor1a^Flx/+^) and Dlx-CKO (Dlx5/6-Cre^+^; Tor1a^Flx/−^) mice were harvested fresh and immediately processed using the FD Rapid Golgi stain kit (FD Neurotechnologies) as per manufacturer’s instructions and as described previously (41). Slides were observed under brightfield microscopy and striatal spiny projection neurons with dense Golgi-cox impregnation without dendritic breaks or obstructions were imaged with a ×63 objective lens and reconstructed using Neurolucida (MBF Bioscience). Spines were assessed on 3rd order or higher dendrites at ≥ 80 μm from the soma. A total of 31 neurons from 6 control animals and 25 neurons from 5 Dlx-CKO animals were assessed.

### Statistics

All data are reported as mean ± SEM unless otherwise indicated. All statistical tests reported (Student’s t-tests, One-way or two-way ANOVAs) were performed using Graphpad Prism (Version 9.3.1).

## Results

### RNA-seq of cholinergic somas and striatal non-cholinergic tissue in maturing striatum

**FIGURE 1:**
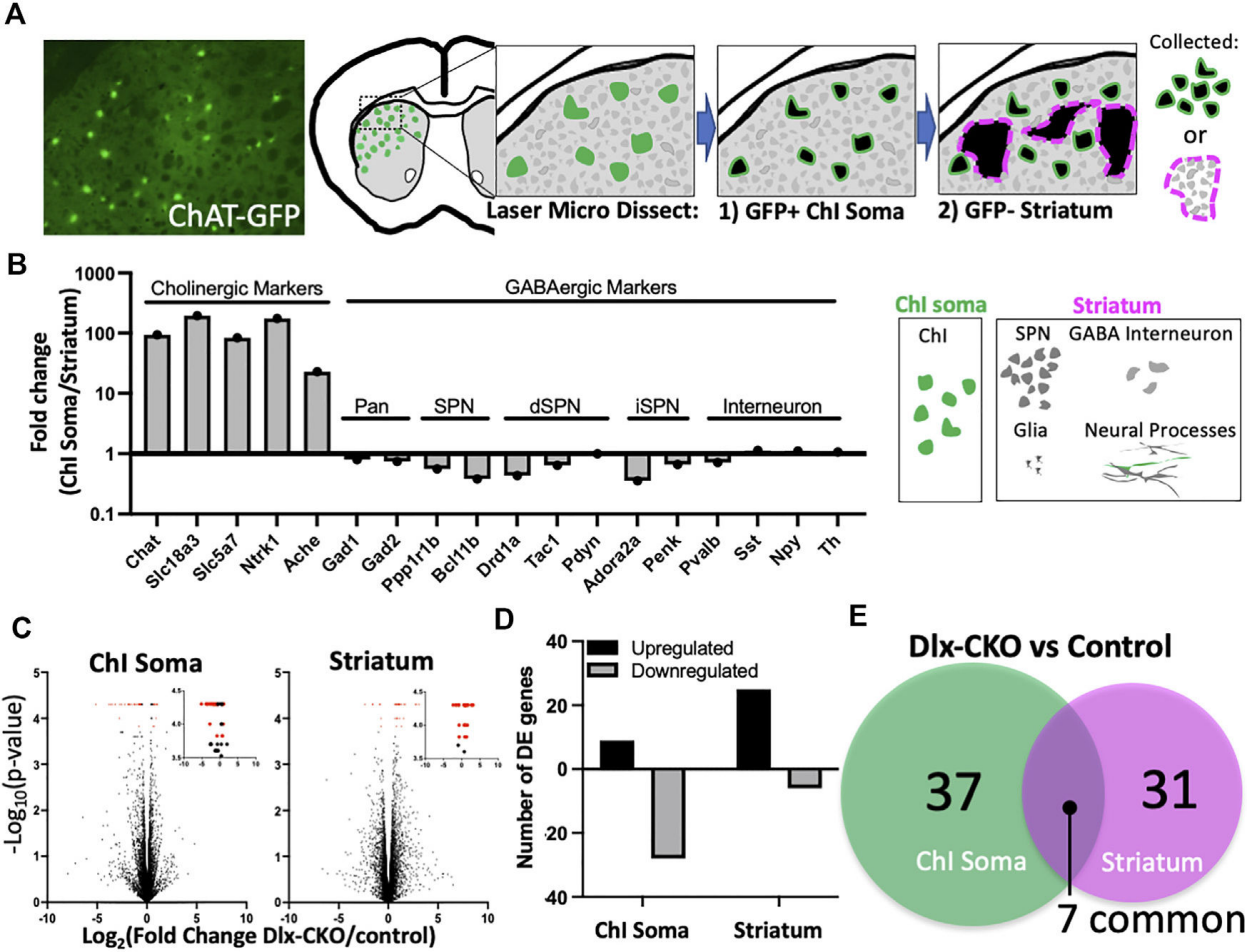
Laser microdissection of cholinergic interneuron somas and non-cholinergic striatum samples identifies differentially expressed genes in Dlx-CKO vs. control genotypes. **(A)** Laser microdissection workflow. ChI somas were dissected from ChAT-GFP+ cells followed by dissection of surrounding GFP-negative striatum comprised mainly of SPN somas, as well as GABA interneuron somas, glia, and neural processes. **(B)** Fold change of cholinergic and GABAergic markers (derived from FPKM) demonstrates enrichment of laser microdissected samples. **(C)** Control vs. Dlx-CKO differentially expressed genes (highlighted in red) identified from RNA-seq analyses (ChI soma samples derived from *n* = 6 control and *n* = 6 Dlx-CKO mice; Striatum samples derived from *n* = 4 controls and *n* = 5 Dlx-CKO mice). Insets show the same data from 3.5–4.5 on the y-axis. All differentially expressed genes are listed in Tables 1, 2. **(D)** Upregulated and downregulated genes from ChI soma and striatum. **(E)** Overlap between ChI soma and striatum differentially expressed genes.

To explore the effects of torsinA loss of function on striatal cholinergic interneurons (ChI) and non-cholinergic cells during development, we performed laser microdissection of dorsolateral striatal Chi somas or surrounding non-cholinergic striatum containing spiny projection neuron cell bodies, interneurons, glia, and neural processes (Figure 1A). We purified total RNA from control (*Tor1a*^*Flx/+*^) and Dlx-CKO (Dlx5/6-Cre^+^; *Tor1a*^*Flx/−*^) ChI soma (*n* = 6 control and *n* = 6 Dlx-CKO) and non-cholinergic striatum (*n* = 4 control and *n* = 5 Dlx-CKO) samples and performed RNA-seq analyses (Methods). ChI soma samples demonstrated up to 193.7-fold higher expression of cholinergic-selective markers compared to striatum samples. Non-cholinergic striatum samples were enriched up to 2.8-fold for GABAergic markers (Figure 1B). Within each sample type, there were not significant differences in the expression of cholinergic or GABAergic markers between control and Dlx-CKO genotypes except for *Pdyn* (Supplementary Table S1). We identified control vs. Dlx-CKO differentially expressed (DE) genes in both ChI soma and striatum samples (Figure 1C, DE genes in red; Methods) after filtering out genes with FPKM values less than 1 in both genotypes (Supplementary Table S2). DE genes were cross referenced with the brainrnaseq.org database of purified cell types (47), which confirmed expected expression levels (FPKM) in the brain. Over 75% of DE genes in ChI somas were downregulated (28/37 genes downregulated), and 80% of DE genes in striatum samples were upregulated (25/31 genes upregulated) (Figure 1D). From these comparisons we identified a core set of 7 genes differentially regulated in both ChI soma and striatum samples (Figure 1E).

### Overrepresentation of differentially expressed genes encoding secreted and extracellular components in ChI soma and non-cholinergic striatum

To assess the functional significance of control vs. Dlx-CKO DE genes, we first determined their subcellular localization by cross referencing with the COMPARTMENTS database (48). Consistent with the role of torsinA in the secretory pathway, 62% of DE genes in ChI soma (23/37 genes) and 42% of DE genes in striatum (13/31 genes) were categorized as secreted, extracellular, extracellular matrix, or plasma membrane localized in mouse (Supplementary Table S3). Similarly, 48% of DE genes in ChI soma (18/37 genes) and 29% of DE in striatum (9/31 genes) are present in the human secretome (49) (Supplementary Table S4).

**FIGURE 2:**
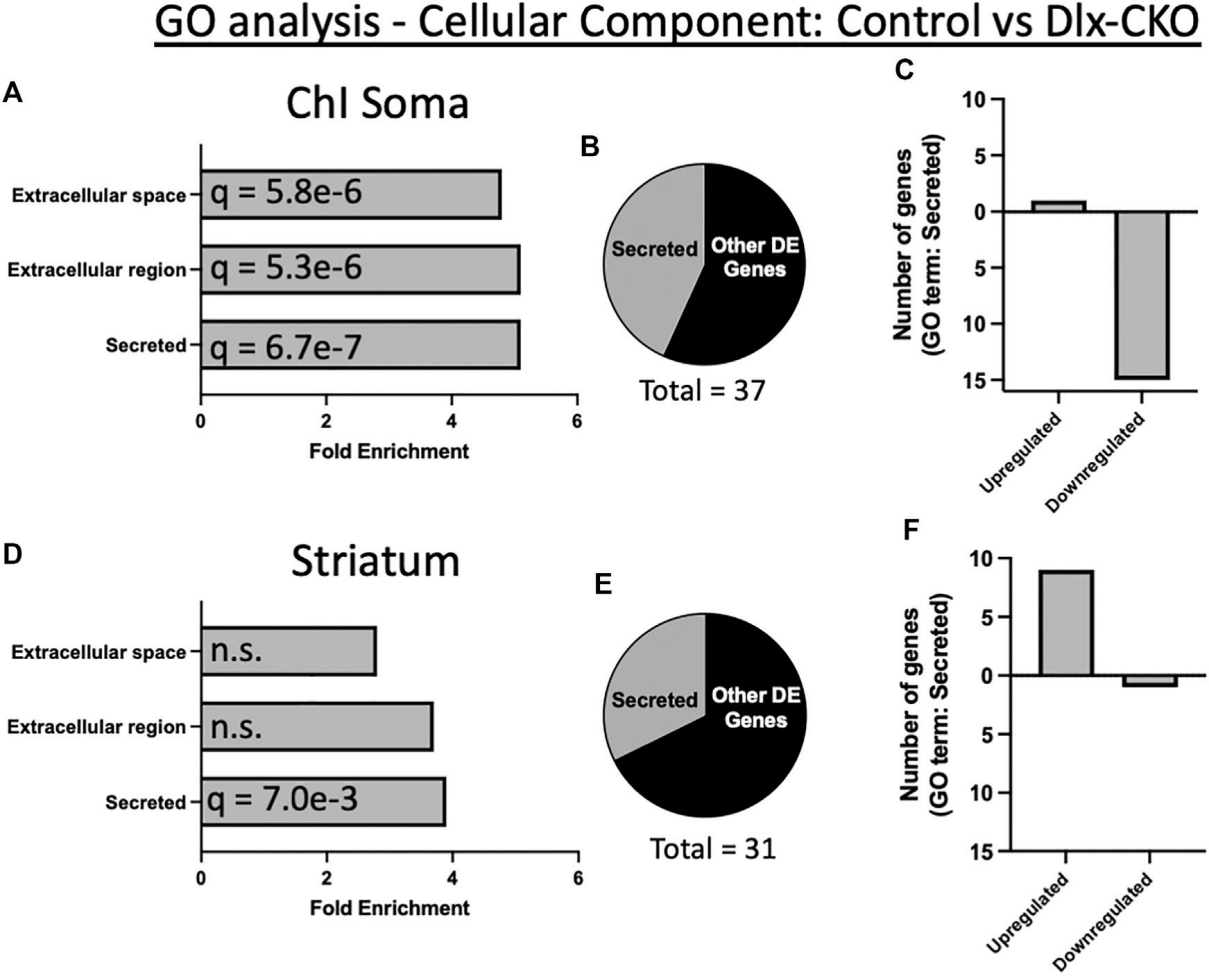
Gene ontology analysis demonstrates over-representation of genes encoding secreted proteins in Dlx-CKO mice. **(A)** A cellular component annotation cluster of secreted, extracellular region, and extracellular space related genes was significantly over-represented in ChI soma samples. **(B)** Percent of all ChI soma differentially expressed genes annotated as secreted. **(C)** Number of upregulated vs. downregulated genes in ChI soma analyses. **(D)** A cellular component annotation cluster of genes encoding secreted proteins was significantly over-represented in non-cholinergic striatum samples. **(E)** Percent of all non-cholinergic striatum differentially expressed genes annotated as secreted. **(F)** Number of upregulated vs. downregulated genes in non-cholinergic striatum samples.

Gene ontology (GO) analysis of *Tor1a* CKO DE genes using DAVID (50) identified a significant over-representation of genes encoding secreted factors in both ChI soma and striatum samples (Supplementary Table S5). An annotation cluster comprising secreted, extracellular region, and extracellular space was significantly over-represented in ChI samples (16/37 genes; cellular component; enrichment score 6.52; Figures 2A, B). Most of these DE genes in ChI were downregulated (Figure 2C). Striatum samples were also over-represented for genes encoding secreted factors (13/31 genes; cellular component; enrichment score 2.07; Figures 2D, E), and most of these DE genes were upregulated (Figure 2F).

### Overrepresentation of synaptic genes in non-cholinergic striatum and dendritic spine alterations in striatal spiny projection neurons

**FIGURE 3:**
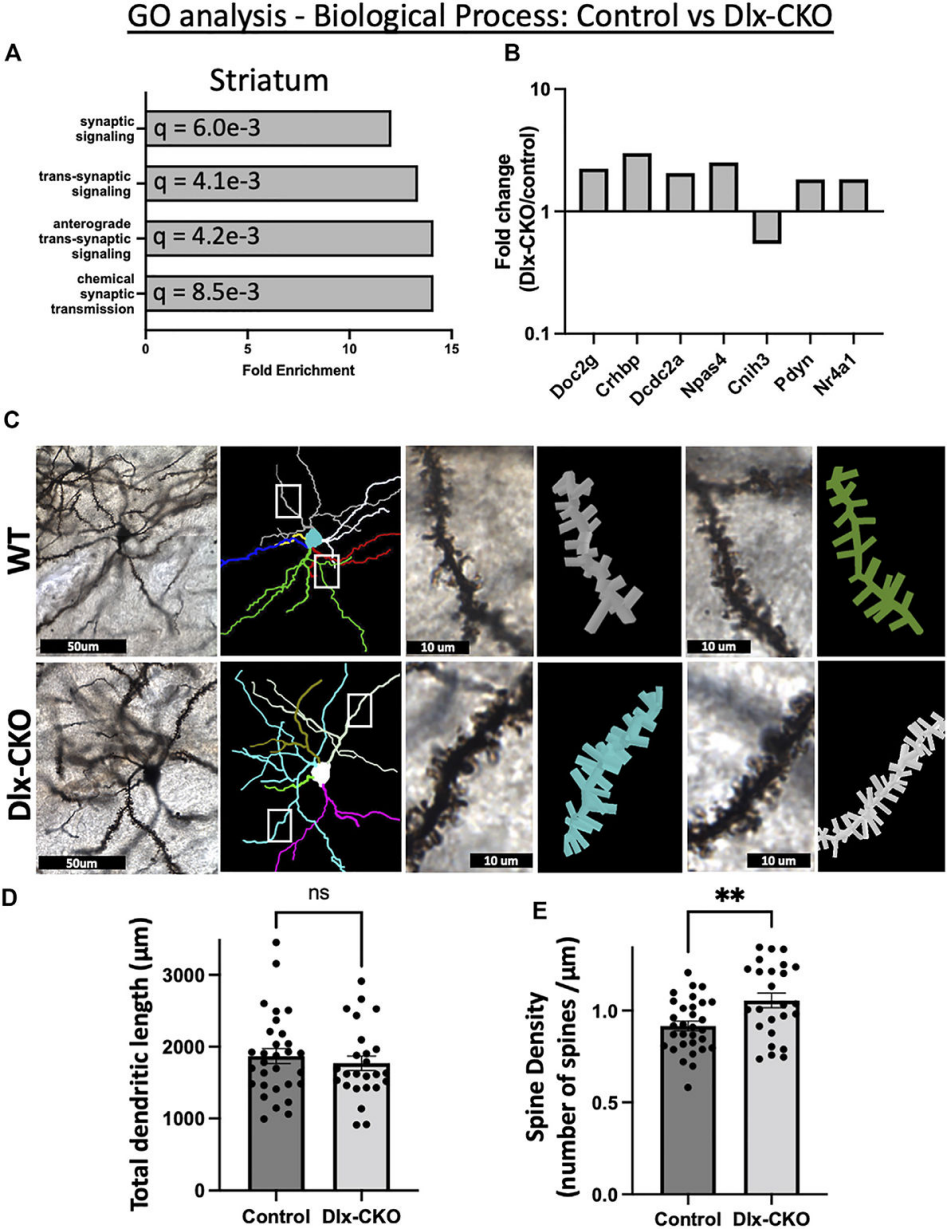
Alterations to synaptic structure and function in Dlx-CKO striatal GABAergic neurons. **(A)** A biological process annotation cluster of synaptic signaling related genes was significantly over-represented in non-cholinergic striatum samples. **(B)** Fold change of the annotated synaptic signaling genes (derived from FPKM). **(C)** Golgi-Cox impregnated striatal spiny projection neurons and associated dendritic arbor reconstructions from control and Dlx-CKO adult mouse brains. **(D)** Total dendritic length of spiny projection neurons (control: *n* = 30 neurons from 6 mice, Dlx-CKO: *n* = 25 neurons from 5 mice. t_53_ = .6718, *p* = .5046). **(E)** Spine density of third order dendrites on spiny projection neurons (control: *n* = 31 neurons from 6 mice, Dlx-CKO: *n* = 25 neurons from 5 mice, t_54_ = 3.008, *p* = .004).

GO analyses (geneontology.org) (51, 52) identified broad changes to synaptic function in non-cholinergic striatum from Dlx-CKO samples as compared to control (Supplementary Table S6). An annotation cluster comprising synaptic signaling, anterograde trans-synaptic signaling, and chemical synaptic transmission was significantly over-represented in striatum samples (Figure 3A). Of the annotated synaptic genes, *Doc2g, Crhbp, Dcdc2a, Npas4, Pdyn*, and *Nr4a1* were upregulated, and *Cnih3* was downregulated (Figure 3B). This cluster of gene expression changes suggests that striatal synaptic structure may be altered in Dlx-CKO mice.

To assess this possibility, we examined dendritic structure in Dlx-CKO and control mice by performing Golgi-Cox impregnation and assessing striatal spiny projection neuron morphology using light microscopy (Figure 3C). Spiny projection neurons are morphologically immature at P14 and their inputs onto dendritic spines continue to mature into adulthood (53-55), so we assessed morphology and spine density in adult brains. Consistent with our previous findings (41), the length of the dendritic arbors of striatal spiny projection neurons were not significantly different between control and Dlx-CKO mice (t_53_ = 0.6718, *p* = 0.5046; Figure 3D). However, the spine density of 3^rd^ order dendritic branches was significantly increased in Dlx-CKO brains compared to control (t_54_ = 3.008, *p* = 0.004; Figure 3D).

Increased spine density reflects increased excitatory input to spiny projection neurons. Consistent with the potential for increased excitability, activity-dependent immediate early genes were significantly upregulated in Dlx-CKO non-cholinergic striatum samples. At least 7 immediate early genes were upregulated in striatum, including *Fos* (1.8 fold), *Arc* (2 fold), *Egr4* (1.8 fold), *Nr4a1* (1.8 fold), *Npas4* (2.5 fold), *Npas2* (2.2 fold), and *Ctgf* (1.85 fold). In ChI samples, *Fos* was significantly upregulated (1.6 fold), suggesting that ChI activity may also be increased in Dlx-CKO mice.

## Discussion

These studies identify a core set of differentially expressed genes in the striatum of torsinA conditional knockout mice during postnatal CNS maturation. Despite the previously reported divergent phenotype between cell types (cholinergic neurodegeneration vs. GABAergic neuron survival (41)), both ChI soma and non-cholinergic striatum samples demonstrated a discrete set of gene expression changes consistent with the role of torsinA in the secretory pathway. Striatum samples also displayed expression changes of genes regulating synaptic transmission and an upregulation of activity-dependent immediate early genes. Consistent with our RNAseq analyses, striatal spiny projection neurons in adult mice demonstrated significantly higher spine density, suggesting that surviving striatal neurons exhibit increased excitability during striatal maturation and increased afferent inputs in adulthood.

We isolated either ChAT-GFP+ ChI somas or GFP negative striatal tissue containing mainly spiny projection neuron somas, as well as GABAergic interneuron somas, glia, and neural projections using laser microdissection (see Figure 1A for a summary of the laser microdissection approach). The ChI soma samples were therefore highly enriched in a single cell type, while striatum samples contained mostly GABAergic neurons in a mixture of cell types and compartments, mainly comprising spiny projection neurons. This is reflected in our analyses as a cholinergic marker enrichment of 193.7 fold vs. GABAergic marker enrichment of up to 2.8 fold. Differential expression analyses of both sample types were overrepresented for genes encoding factors that are secreted to the extracellular space. Several neuropeptides were overrepresented in Dlx-CKO ChI soma samples, including Pdyn (upregulated), Vip, Npy, Cartpt, and Sst (downregulated). These factors were previously found to be enriched in GABAergic striatal neurons (56-61), but our enrichment protocol may have enabled measurement of sparse neuropeptide expression. The differential expression of genes encoding extracellular proteins and neuropeptides are consistent with a central role of torsinA in the secretory pathway (21, 22), as suggested by its localization in the endoplasmic reticulum lumen (3, 62).

Dlx-CKO striatum samples demonstrated a suite of gene expression differences consistent with a structural or functional change in striatal synapses. Whether the synaptic changes of GABAergic neurons reflect intrinsic responses to torsinA deficiency or a compensation consequent to neighboring cholinergic neurodegeneration remains unknown. Striatal cholinergic signaling matures postnatally and begins to dynamically regulate the synaptic activity of other striatal neurons as skilled motor function develops (63). The second postnatal week (when samples were collected in this study) is a maturational period during which corticostriatal synaptogenesis and spinogenesis begins and progresses (reviewed in (64)) as activity induced factors shape the connectivity of striatal neurons (65, 66). Several differentially expressed genes identified in this study modulate striatal spiny projection neuron spine density. The nuclear receptor *Nr4a1* (upregulated 1.83 fold in Dlx-CKO) is enriched in spiny projection neurons (67), where its activity-induced expression alters spine density as part of a transcriptional program that regulates density and distribution of dendritic spines (68, 69) and promotes spiny projection neuron maturation (67). *Npas4* (upregulated 2.51 fold) is a transcription factor that regulates GABAergic synaptic function (70) and is important for synaptic formation, function and ongoing plasticity (71). Knockdown of *Npas4* reduces dendritic spine density on D1 receptor-expressing spiny projection neurons (72). Expression of the cytoskeleton associated protein Arc (upregulated 2-fold in Dlx-CKO striatum) increases spine density *in vivo* (73, 74). IGF-1 (upregulated 2.19-fold in Dlx-CKO striatum) administration rescues spine density (75) or spine motility (76) in Mecp2 mutant mice and knockdown of IGF-1 decreases spine density of purkinje cells (77). The upregulation of these factors during striatal maturation is consistent with changes to synaptic structure, as evidenced by significantly increased spine density of Golgi-Cox-stained spiny projection neurons in the present study.

Our differential expression analyses also suggest functional synaptic changes in Dlx-CKO mice. *Doc2g* (upregulated 2.24 fold in Dlx-CKO) is a member of the DOC2 family of proteins that modulates spontaneous synaptic transmission (78). Knockdown of DOC2 proteins triggers excitatory synaptic scaling without altering action potential dependent activity (79). *Cnih3* (downregulated 1.84 fold in Dlx-CKO) is an AMPA receptor auxiliary subunit that functions in the endoplasmic reticulum and remains associated with the AMPA receptor complex at the synapse (80). CNIH3 regulates AMPA receptor trafficking and gating properties by determining the subunit composition of heteromeric AMPA receptors (81) and controlling the export of AMPA receptors from the endoplasmic reticulum (82). The structure of the interface between CNIH3 and AMPA receptors suggests that lipids play a role in the assembly of these complexes (83). The endoplasmic reticulum localization of CNIH3 and its interplay with lipids in complex with AMPA receptors suggests that it could be one link between torsinA function and the synaptic plasticity differences observed in animal models (24,35-37) and in people with dystonia (31-34). The synapse-related gene expression changes identified in torsinA null striatal neurons during maturation may therefore contribute to long lasting enhancement of spiny projection neuron synaptic structure and function.

To our knowledge, spine density has not been assessed previously in torsinA null mice. Heterozygous *Tor1a*^*ΔE/+*^ mice have reduced spiny projection neuron spine density at P26 (29), but no difference at P60 (29), consistent with other spine density studies in adult *Tor1a*^*ΔE/+*^ mice (27, 84). Spine density on distal dendrites of cerebellar purkinje neurons is reduced in 3 month old *Tor1a*^*ΔE/+*^ animals (28). However, motor behavior is not altered in these mice (85).

Surprisingly, despite glial enrichment, Gfap (encoding Glial Fibrillary Acidic Protein) was upregulated in both ChI soma and non-cholinergic striatum samples of Dlx-CKO mice. ChI soma sample *Gfap* expression could reflect “contamination” with adjacent or (synapsed) astrocytes, as increased neuronal activity increases expression of glial *Gfap* (86). However, astrogliosis is not observed in Dlx-CKO striatum (41). Neurons can express Gfap in neurodegenerative disease (87), but we observed robust Gfap expression in both control and Dlx-CKO samples. Some neuronal *Gfap* expression is observed in the normal mouse brain ((47); brainrnaseq.org). Fate mapping studies demonstrate that *Gfap*-expressing progenitors give rise to some neurons, including in the striatum (88), suggesting that we may be observing physiological ChI expression of *Gfap* during striatal maturation.

Six genes were differentially expressed in *both* ChI soma and non-cholinergic striatum samples. *Fos, Pdlim3*, and *Pdyn* were all upregulated to similar extents in both sample types, suggesting that these genes could represent common responses to torsinA loss of function or striatal circuit changes. In contrast, *Ptgds, Tuba1c*, and *Gfap* were downregulated in ChI somas, but upregulated in non-cholinergic striatum, suggesting a role in differential vulnerability of striatal neurons to cell death or cell type specific responses to torsinA loss of function. *Tuba1c* reduction (35.71 fold decreased in ChI) may reflect microtubule disruption or active degeneration of ChI, while its increase in non-cholinergic striatum (6.98 fold increased) could reflect compensatory neurite outgrowth or axon elongation in surviving cells (89). Only a single tubulin isoform was altered in this study, suggesting that torsinA loss of function caused a highly specific change rather than broad disruption of microtubule structure. Microtubule dynamics contribute to dendritic spine development, morphology, and synaptic plasticity (90-93). Increased *Tuba1c* expression may therefore reflect or contribute to the spine density increases we observed in Dlx-CKO spiny projection neurons.

*Ptgds* encodes lipocalin type prostaglandin D2 synthase, which catalyzes the conversion of prostaglandin H2 to the neuromodulatory prostaglandin D2 in the brain (94-96). Prostaglandin D2 is neuroprotective in contexts such as hypoxia-ischemic injuries, excitotoxicity, and oxidative stress (97,98,99,100,101). Prostaglandin D2 synthase (also called β-trace) itself is a neuroprotective chaperone that inhibits Aβ aggregation (102, 103), and alterations to its expression may be a biomarker of several neurological disorders (104). In the present study, *Ptgds* was 14.08 fold decreased in ChI soma and 6.56 fold increased in non-cholinergic striatum. Ptgds upregulation could contribute to the selective survival of non-cholinergic neurons in the striatum of Dlx-CKO mice. Further investigations would be required to determine whether this association is causative.

This study supports a developmental role for torsinA in the secretory pathway and demonstrates abnormal synaptic development in the torsinA deficient striatum. These transcriptomic datasets are freely available as a resource for future hypothesis driven work exploring the consequences of torsinA loss for striatal structure and function.

## Supplementary Material

Table S1 **SUPPLEMENTARY TABLE S1** Cell type markers control vs. Dlx-CKO (internal control).

Table S2 **SUPPLEMENTARY TABLE S2** Filtered genes removed from analysis.

Table S3 **SUPPLEMENTARY TABLE S3** Cross reference with the COMPARTMENTS database.

Table S4 **SUPPLEMENTARY TABLE S4** Cross reference with the Human Secretome.

Table S5 **SUPPLEMENTARY TABLE S5** Gene ontology analysis using DAVID: over-representation of genes encoding secreted proteins.

Table S6 **SUPPLEMENTARY TABLE S6** Gene ontology analysis using geneontology.org: over-representation of genes encoding proteins regulating synaptic structure and function.

Table S7 **SUPPLEMENTARY TABLE S7** ChI soma raw count data matrix.

Table S8 **SUPPLEMENTARY TABLE S8** Striatum raw count data matrix.

Table S9 **SUPPLEMENTARY TABLE S9** Aligned reads for each sample.

## Figures and Tables

**TABLE 1:** Differentially Expressed genes from striatal cholinergic interneuron soma samples.

Gene	Gene ID	Description	Control FPKM	Dlx-CKO FPKM	Fold change	q_value
Pdlim3	53318	PDZ and LIM domain 3	6.324	12.967	2.05	.01740
Rbm45	241490	RNA binding motif protein 45	7.387	14.970	2.03	.01740
Hmox1	15368	heme oxygenase 1	3.711	7.056	1.90	.03139
Cd59a	12509	CD59a antigen	14.549	26.403	1.81	.01740
Pdyn	18610	Prodynorphin	22.559	39.175	1.74	.01740
Prr5l	72446	proline rich 5 like	1.567	2.589	1.65	.04447
Fos	14281	FBJ osteosarcoma oncogene	7.107	11.580	1.63	.01740
Sdf2l1	64136	stromal cell-derived factor 2-like 1	31.479	49.964	1.59	.01740
Itga9	104099	integrin alpha 9	2.025	3.135	1.55	.04447
Grtp1	66790	GH regulated TBC protein 1	30.451	20.193	.66	.01740
Npy	109648	neuropeptide Y	609.880	401.328	.66	.01740
Sv2c	75209	synaptic vesicle glycoprotein 2c	11.522	7.481	.65	.01740
Cartpt	27220	CART prepropeptide	69.853	43.915	.63	.01740
Sst	20604	Somatostatin	1,044.770	646.451	.62	.01740
Beta-s	100503605	hemoglobin, beta adult s chain	351.612	213.052	.61	.01740
Col1a2	12843	collagen, type I, alpha 2	3.317	1.927	.58	.01740
F2r	14062	coagulation factor II (thrombin) receptor	2.813	1.626	.58	.04447
Hba-a2	110257	hemoglobin alpha, adult chain 2	185.868	107.032	.58	.01740
Igfbp2	16008	insulin-like growth factor binding protein 2	30.841	17.678	.57	.01740
Hddc3	68695	HD domain containing 3	47.292	26.738	.57	.01740
Insrr	23920	insulin receptor-related receptor	3.712	2.047	.55	.01740
Gfap	14580	glial fibrillary acidic protein	36.399	17.785	.49	.01740
Serping1	12258	serine (or cysteine) peptidase inhibitor, clade G, member 1	5.829	2.531	.43	.01740
Igf2	16002	insulin-like growth factor 2	6.092	2.557	.42	.01740
Gjb2	14619	gap junction protein, beta 2	2.135	.887	.42	.01740
Col1a1	12842	collagen, type I, alpha 1	1.983	.690	.35	.01740
Nov	18133	nephroblastoma overexpressed gene	4.641	1.409	.30	.01740
spp1	20750	secreted phosphoprotein 1	3.096	.907	.29	.01740
Dcn	13179	Decorin	5.817	1.624	.28	.01740
Slc6a13	14412	solute carrier family 6 (neurotransmitter transporter, GABA), member 13	2.547	.686	.27	.01740
Fam180a	208164	family with sequence similarity 180, member A	2.217	.384	.17	.01740
Vip	22353	vasoactive intestinal polypeptide	2.470	.374	.15	.01740
Fmod	14264	Fibromodulin	2.703	.293	.11	.01740
Aldh1a2	19378	aldehyde dehydrogenase family 1, subfamily A2	2.253	.205	.09	.01740
Slc13a4	243755	solute carrier family 13 (sodium/sulfate symporters), member 4	2.153	.170	.08	.01740
Ptgds	19215	prostaglandin D2 synthase (brain)	609.567	43.543	.07	.01740
Tuba1c	22146	tubulin, alpha 1C	14.184	.407	.03	.01740
Tor1a	30931	torsin family 1, member A (torsin A)	47.518	10.149	.21	.01740

**TABLE 2:** Differentially Expressed genes from non-cholinergic striatum samples.

Gene	Gene ID	Description	Control FPKM	Dlx-CKO FPKM	Fold change	q_value
Eln	13717	Elastin	1.577	13.526	8.58	.01960
Tuba1c	22146	tubulin, alpha 1C	.624	4.356	6.98	.01960
Ptgds	19215	prostaglandin D2 synthase (brain)	2.139	14.035	6.56	.01960
Serpina3n	20716	serine (or cysteine) peptidase inhibitor, clade A, member 3N	1.554	5.061	3.26	.01960
Crhbp	12919	corticotropin releasing hormone binding protein	1.592	4.775	3.00	.03323
Pdlim3	53318	PDZ and LIM domain 3	6.686	19.440	2.91	.01960
Npas4	225872	neuronal PAS domain protein 4	1.169	2.943	2.52	.04679
Gadd45g	23882	growth arrest and DNA-damage-inducible 45 gamma	24.837	62.021	2.50	.01960
Doc2g	60425	double C2, gamma	3.626	8.125	2.24	.03323
Igf1	16000	insulin-like growth factor 1	.655	1.438	2.20	.01960
Npas2	18143	neuronal PAS domain protein 2	4.878	10.687	2.19	.01960
Crip1	12925	cysteine-rich protein 1 (intestinal)	27.202	58.769	2.16	.01960
Dcdc2a	195208	doublecortin domain containing 2a	.946	1.942	2.05	.01960
Arc	11838	activity regulated cytoskeletal-associated protein	44.295	89.269	2.02	.01960
Hspb1	15507	heat shock protein 1	13.250	25.970	1.96	.03323
Ctgf	14219	connective tissue growth factor	3.671	6.825	1.86	.03323
Nr4a1	15370	nuclear receptor subfamily 4, group A, member 1	51.004	93.507	1.83	.01960
Pdyn	18610	Prodynorphin	21.950	39.961	1.82	.03323
Fos	14281	FBJ osteosarcoma oncogene	7.206	12.946	1.80	.04679
Rbp4	19662	retinol binding protein 4, plasma	19.486	34.677	1.78	.01960
Egr4	13656	early growth response 4	97.187	172.884	1.78	.01960
Gfap	14580	glial fibrillary acidic protein	19.204	32.604	1.70	.01960
Mgp	17313	matrix Gla protein	55.013	92.441	1.68	.03323
Rps21	66481	ribosomal protein S21	899.763	1,416.980	1.57	.01960
Sec61b	66212	Sec61 beta subunit	197.548	309.101	1.56	.04679
Etl4	208618	enhancer trap locus 4	5.189	3.150	.61	.03323
Cdr1	631990	cerebellar degeneration related antigen 1	103.307	62.310	.60	.01960
Gpx6	75512	glutathione peroxidase 6	27.586	15.782	.57	.01960
Cnih3	72978	cornichon family AMPA receptor auxiliary protein 3	23.884	12.954	.54	.01960
Xist	213742	inactive X specific transcripts	5.991	2.035	.34	.01960
Pla2g4e	329502	phospholipase A2, group IVE	2.525	.854	.34	.01960
Tor1a	30931	torsin family 1, member A (torsin A)	34.565	6.742	.20	.01960

## Data Availability

All raw RNAseq data from this study is included as Supplementary Material and all differentially expressed genes are listed within the main article. Further inquiries can be directed to the corresponding author.
